# Thio Derivatives of 2(5H)-Furanone as Inhibitors against Bacillus subtilis Biofilms

**Published:** 2015

**Authors:** E. Yu. Trizna, E. N. Khakimullina, L. Z. Latypova, A. R. Kurbangalieva, I. S. Sharafutdinov, V. G. Evtyugin, E. V. Babynin, M. I. Bogachev, A. R. Kayumov

**Affiliations:** Kazan (Volga Region) Federal University, Kremlevskaya Str., 18, 420008, Kazan, Russia; St. Petersburg State Electrotechnical University, Prof. Popova Str., 5, 197376, St. Petersburg, Russia

**Keywords:** antibacterial activity, biofilms, 2(5H)-furanones, Bacillus subtilis

## Abstract

Gram-positive bacteria cause a wide spectrum of infectious diseases, including nosocomial infections. While in the biofilm, bacteria exhibit increased resistance to antibiotics and the human immune system, causing difficulties in treatment. Thus, the development of biofilm formation inhibitors is a great challenge in pharmacology. The gram-positive bacterium *Bacillus subtilis *is widely used as a model organism for studying biofilm formation. Here, we report on the effect of new synthesized 2(5*H*)-furanones on the biofilm formation by *B.subtilis *cells. Among 57 compounds tested, sulfur-containing derivatives of 2(5*H*)-furanone (F12, F15, and F94) repressed biofilm formation at a concentration of 10 μg/ml. Derivatives F12 and F94 were found to inhibit the biosynthesis of GFP from the promoter of the *eps *operon encoding genes of the biofilm exopolysaccharide synthesis (EPS). Using the differential fluorescence staining of alive/dead cells, we demonstrated an increased bacterial sensitivity to antibiotics (kanamycin and chloramphenicol) in the presence of F12, F15, and F94, with F12 being the most efficient one. The derivative F15 was capable of disrupting an already formed biofilm and thereby increasing the efficiency of antibiotics.

## INTRODUCTION

It has now been established that in nature most bacteria exist in the form of specifically organized biofilms. Biofilms are a community of differentiated microbial cells tightly adhered to a substrate that are embedded in a polysaccharide matrix (EPS). This form of existence provides bacteria with a series of advantages under the influence of negative environmental factors and of the host organism. This leads, on one hand, to an increased efficiency of biotechnological processes and, on the other hand, to enhanced resistance to antimicrobial agents, antiseptics and disinfectants, and refractoriness to treatment, which results in an increased incidence of nosocomial infections and creates difficulties in microbiological diagnostics of infectious diseases [1-3]. Therefore, biofilms represent a serious problem and require the development of drugs that disrupt bacterial biofilms and inhibit their formation on medical devices.* Bacilli*, gram-positive spore-forming rods, e.g., *Bacillus anthracis *and *Bacillus cereus*, which cause anthrax and severe foodborne toxicoinfections, also form biofilms on various surfaces [1]. *B. subtilis *cells are widely used as a model for studying bacillus biofilms [1].

Nowadays, bacterial biofilms are treated by coating surfaces with silver particles, immobilized enzymes disrupting the biofilm matrix, as well as various lowmolecular weight substances that act as inhibitors of biofilm formation genes [4]. Among these substances, a special place belongs to compounds of the 2(*5H*)-furanone series [5] that were firstly isolated from the red alga *Delisea pulchra. *Furanone derivatives have been shown to possess antimicrobial activity against a great number of gram-positive and gram-negative bacteria and inhibit biofilm formation [5, 6].

## EXPERIMENTAL

**Furanones**

**Fig. 1:**
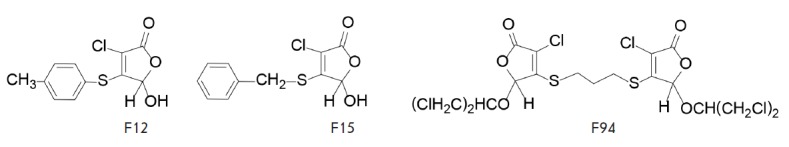
Structures of furanones that inhibit *B.subtilis *biofilm formation at a concentration of 10 μg/ml

*Fig. 1* depicts the structures of the studied compounds: F12 – 5-hydroxy-4-[(4-methylphenyl)sulfonyl]- 3-chloro-2(*5H*)-furanone [7], F15 – 4-benzyl-sulfonyl- 5-hydroxy-3-chloro-2(*5H*)-furanone [8], and F94 – 1,3-*bis*[3-chloro-5-(1,3-dichloropropane-2-yloxy)- 2(*5H*)-furanone-4-ylsulfonyl] propane [9]; the compounds were synthesized according to the known techniques.

**Strains and culture conditions**

The following strains were used in the study: *B. subtilis* 168 [10]; *B. subtilis *K511 [11] carrying the *gfp *gene under the control of the promoter of the *epsA *gene, which is active during biofilm formation in *B.subtilis*.

The strains *Salmonella typhimurium *TA100 (*HisG46, rfa, uvr-, pkm 101, bio-*) [12] and *S. typhimurium* TA1535/pSK1002 [13] were used to test the compounds for mutagenicity.

All the bacterial strains were maintained and cultured in a LB medium (1.0 g/L of tripton; 0.5 g/L of yeast extract; 0.5 g/L of NaCl; pH 8.5) [14]. Biofilm formation was determined using a BM medium (Basal medium), which is a modified SMM medium [15] supplemented with peptone to a final concentration of 7 g/L.

**Biofilm staining with crystal violet**

Biofilm formation was assessed in 96-well plastic plates (Cellstar Grenier bio-one No. 655 180) by staining with crystal violet. Bacteria were cultured in BM at 37 oC without shaking in wells containing 200 µl of the bacterial culture with an initial density of 3 × 10^7^ CFU/ml. After 72 h of incubation, the culture liquid was removed and the plates were washed once with phosphate-buffered saline (PBS) pH 7.4 and dried for 20 min. Then, 150 µl of a 0.1% crystal violet solution (Sigma-Aldrich) in 96% ethanol was added per well and the plates were further incubated for 20 min. The unbounded dye was washed off with PBS. The bound dye was eluted in 150 µl of 96% ethanol, and the absorbance at 570 nm was measured on a Tecan Infinite 200 Pro microplate reader (Switzerland). Cell-free wells that were subjected to all staining manipulations were used as a control.

**Determination of the minimum inhibitory concentration**

The minimum inhibitory concentration (MIC) of furanones was determined by broth microdilution method in the BM medium in 96-well plastic plates. The concentrations of furanones after serial dilutions were in the range of 0.1–500 µg/µl. The wells were seeded with 200 ml of the bacterial culture (3 × 10^7^ CFU/ml) in the BM medium and incubated at 37 °C. The minimum inhibitory concentration was determined as the lowest concentration of furanone for which no visible bacterial growth was observed after 24 h of incubation. The minimum biofilm inhibitory concentration (MBIC) was determined as the lowest concentration of furanone that completely inhibited biofilm formation after 72 h of growth.

**Determination of the geno- and cytotoxicity of furanones**

The mutagenicity of furanones at the MBIC concentration was evaluated in the Ames test [12]. We used the dimethyl sulfoxide (DMSO) solvent as a negative control and sodium azide (NaN_3_) as a positive control. A tested compound was considered to be mutagenic if the number of revertant colonies in the experiment was more than 2 times higher than that in the control (solvent). The DNA-damaging activity of the compounds was evaluated in the SOS chromotest using the *S. typhimurium* TA1535/pSK1002 strain [13]. The overnight bacterial culture was diluted 10 times with a LB medium and grown in the presence of the study compounds for 4 h. Next, the cells were collected by centrifuging and the β-galactosidase activity was determined according to [16]. Cytotoxicity of the compounds was determined using the MTS test (Promega) on MCF-7 cells, and the median cytotoxicity concentration CC_50_ (the concentration required to reduce cell activity by 50%) was calculated.

## RESULTS AND DISCUSSION

Earlier, we identified halogen- and sulfur-containing derivatives of 2(*5H*)-furanone that inhibited* B. subtilis *biofilm formation [6]. Additional screening of 56 substances enabled the identification of two more furanones (F15 and F94) inhibiting the biofilm formation at a concentration of 10 µKg/ml (*Table 1*). F2 and F8 (5-hydroxy-4-[(4-methylphenyl) sulfonyl]-3-chloro-2(*5H*)-furanone and 3,4-dichloro- 5-[(1,3-dichloropropane-2-yloxy)]-2(*5H*)-furanone, respectively), which were characterized in reference [6], increased the activity of the genetic competence system of *B. subtilis *and were not included in further research. F15 and F94 did not increase the activity of the transcription factor ComA, which activates the system of genetic competence development in *Bacilli* (not shown).

**Table 1:** Minimum furanone concentrations inhibiting B.subtilis 168 growth and biofilm formation; cyto- and genotoxic properties of the compounds

Furanone	Minimum inhibitory concentration (MIC), µg/ml	Minimum biofilm inhibitory concentration (MBIC), µg/ml	CC_50_ for MCF-7 cells, µg/ml	Genotoxicity of compounds (excess over the control, times/cell number)	
				Ames test*	SOS chromotest*
F12	25	10	36.9	2.4 (109 ± 25.2)	0.69
F15	25	10	65.7	3.1 (133 ± 25.4)	0.61
F94	50	10	83.9	0.9 (41 ± 4.4)	1.09
Control**	-	-	-	1.0 (45 ± 3.5)	1.00
Positive control***	-	-	-	8.2 (369 ± 15.6)	22.71

* Genotoxicity was evaluated at a 10 µg/ml concentration of furanones (corresponds to their MBIC values).

** Amount of dimethyl sulfoxide added in the form of a furanone solution.

*** Sodium azide (3 µg/ml) and mytomycin C (0.1 µg/ml) were used in the Ames test and SOS chromotest, respectively.

**Fig. 2:**
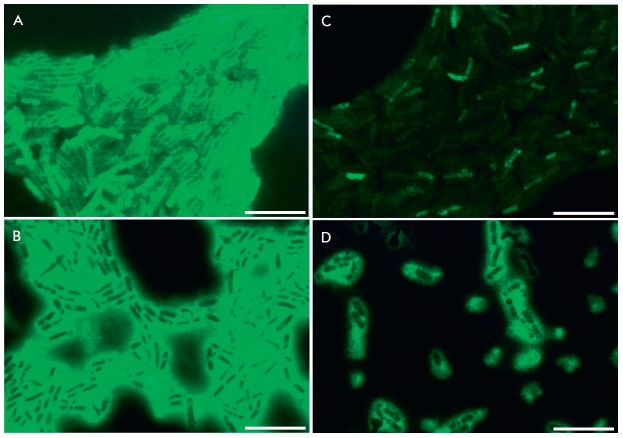
The effect of furanones on the fluorescence intensity of GFP expressed from the *eps *operon promoter in* B. subtilis *K511 cells. Cells were grown in the presence of F12 (*B*), F15 (*C*), and F94 (*D*) at a concentration of 10 µg/ml (corresponds to MBIC) for 72 h. Cells grown in the absence of furanones were used as a control (*A*). The scale bar is 10 µm

In order to establish the influence of furanones on the expression level of the *eps *operon encoding biofilm EPS synthesis genes, *B. subtilis *K511 cells carrying the *gfp *gene under control of the *epsA *gene promoter were grown in a BM medium in the presence/ absence of furanones for 72 h and analyzed using a fluorescent microscope (*Fig. 2*). Detection of GFP in the cells in the absence of furanones indicated expression of the *eps *operon and production of EPS, which is the biofilm matrix basis (*Fig. 2A*). GFP was not identified in the presence of furanones F12 and F94, suggesting the repression of EPS production and, as a consequence, the repression of biolfilm formation in the presence of these compounds (*Fig. 2 B,D*). Apparently, the molecular targets for these compounds are the regulatory pathways of organism adaptation to stress conditions. Indeed, F12 was demonstrated to inhibit the activity of the transcription factors Spo0A and TnrA [6]. On the contrary, GFP was also detected in the presence of F15, although in substantially lower amounts compared to the control; therefore, no suppression of the *eps *operon occurred. It is possible that F15 inhibits the biofilm formation through a different pathway, without involvement of the *eps *operon regulation.

**Furanones increase the sensitivity of adhered cells to antibiotics**

Antimicrobial agents are known to be ineffective against bacteria in the biofilm mode of existence. Presumably, the repression of biofilm formation should increase the efficiency of antimicrobial agents. Potential synergism between furanones and antibiotics was studied using the chessboard method, where the furanone and antibiotic (kanamycin and chloramphenicol) concentrations were varied from 0.1 to 2.0 MIC [17]. However, no compound exhibited synergism with antimicrobial agents with respect to plankton cells (FIC = 1.2 ± 0.21).

**Fig. 3:**
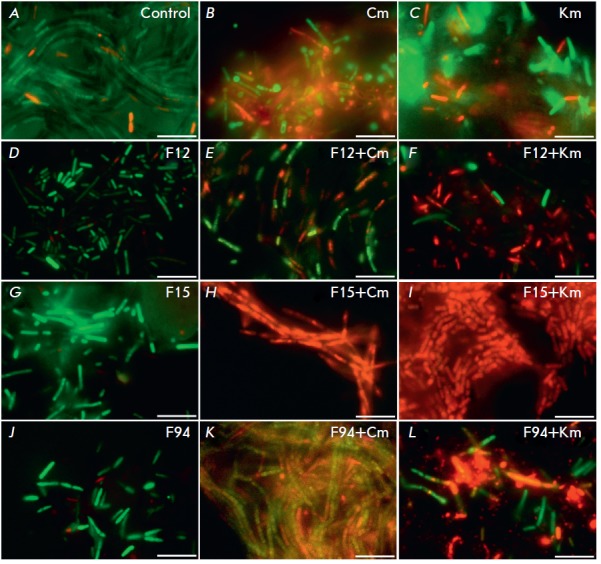
The effect of furanones on biofilm formation by *B.subtilis* cells and the sensitivity of *B.subtilis *cells adhered to the culture plate surface to antibiotics.* B.subtilis *168 cells were grown for 72 h to form a biofilm in the absence (*A, B, C*) or presence of furanones (*D, G, J*) at a concentration of 10 µg/ml (corresponds to MBIC). Then, chloramphenicol (Cm) (*E, H, K*) or kanamycin (Km) (*F, I, L*) was added. After 24 h of incubation with an antibiotic, the number of viable cells was analyzed by staining the cells with propidium iodide and fluorescein diacetate. The scale bar is 10 µm

In order to investigate whether furanones increase the sensitivity of surface-adhered bacteria to antibiotics,* Bacilli *were grown in a BM medium in the presence of furanones at a 10 µKg/ml concentration (MBIC) for 72 h, then antibiotics (chloramphenicol and kanamycin) were added to a final concentration of 10 µKg/ml (established MIC values were 2.5 µKg/ml). After 24 h of cultivation, the culture liquid was removed, the biofilm was washed once with PBS, and differential fluorescent staining with propidium iodide and fluorescein diacetate was performed to identify dead and alive cells, respectively, in the layer of microbial cells adhered to the culture plate surface. The obtained specimens were analyzed using a Carl Zeiss Axio Imager 2.0 fluorescent microscope (*Fig. 3*).

**Table 2:** The effect of furanones on the thickness of a B. *subtilis biofilm*

Furanone	Biofilm thickness, µm	
	Cultivation with preliminary added furanones, 96 h	Addition of furanones to the formed biofilm with further incubation for 24 h
Control	10 ± 1.6	10 ± 1.3
F12	4 ± 0.4	6 ± 0.3
F15	2 ± 0.3	4 ± 0.2
F94	4 ± 0.6	8 ± 0.7

The formation of a biofilm up to 10 µm thick was observed in the control sample (*Fig. 3A*, (*Table 2*). In this case, addition of chloramphenicol (*Fig. 3B*) or kanamycin (*Fig. 3C*) resulted in the death of only a small fraction of the adhered cells. In contrast, in the culture grown in the presence of F15 (10 µm/ml), the biofilm thickness was 2 µm, and addition of an antibiotic resulted in almost complete death of bacilli (*Fig. 3 H,I*), while furanone itself had no bactericidal effect (*Fig. 3G*). In case of F12 and F94 at the concentration of 10 µKg/ml, the effect was less pronounced. Thus, the presence of furanones in the culture medium inhibited biofilm formation on the culture dish surface and increased the efficiency of the antibiotics, apparently due to a longer exposure of bacterial cells to antimicrobial agents.

**Fig. 4:**
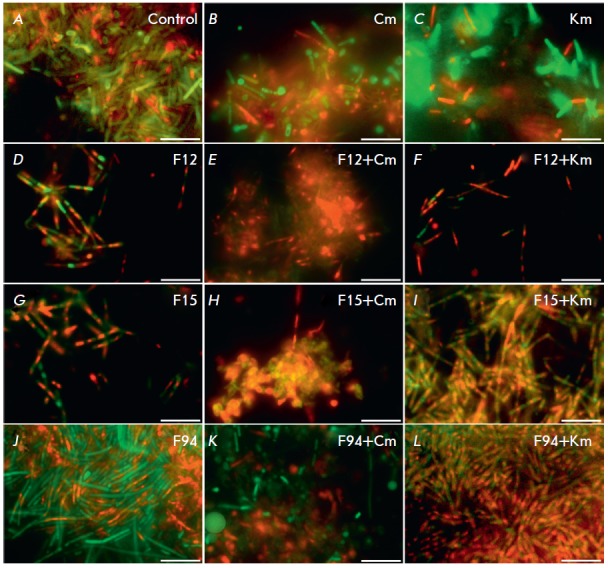
Furanones disrupt a biofilm and increase the efficiency of antibiotics against biofilm-embedded* B.subtilis *cells. *B.subtilis* cells were cultured for 72 h to form a biofilm (*A, B, C*). Then, furanones were added to a final concentration of 30 µg/ml (threefold excess of MBIC) (*D, G, J*) in the presence of chloramphenicol (Cm) (*E, H, K*) or kanamycin (Km) (*F, I, L*). After 24 h of incubation with an antibiotic, the number of viable cells was analyzed by staining the cells with propidium iodide and fluorescein diacetate. The scale bar is 10 µm

The possibility of bacterial biofilm disruption in the presence of furanones was also studied. For this purpose, we grew *B. subtilis *cells in a BM medium for 72 h, removed the culture liquid, and added a pure BM medium supplemented with furanones (30 µKg/ml), kanamycin, and chloramphenicol. After 24 h, the residual biofilm was washed with PBS and differential fluorescent staining was performed (*Fig*. *4*).

As in the previous experiment, antibiotics in the absence of furanones were found to be ineffective against the cells embedded in the biofilm matrix (*Fig. 4 B,C*). Supplementation with F12 (30 µKg/ml) caused significant biofilm disruption after 24 h (*Table 2*), and addition of antibiotics caused the death of the vast majority of cells (*Fig. 4 D-F*). In this case, the effect of F15 was less pronounced, while F94 caused almost no increase in the sensitivity of the cells to the antibiotics and did not lead to biofilm disruption (*Fig. 3 G-L*).

**Cyto- and genotoxic properties of compounds F12, F15, and F94**

Determination of the cytotoxicity of F12, F15, and F94 showed that their CC_50_ values were 7 times higher than the concentrations necessary to inhibit biofilm formation (*Table 1*). Although the SOS chromotest did not detect the DNA damaging activity of the compounds, the Ames test data indicated potential mutagenicity of F12 and F15.

## CONCLUSIONS

Thus, the thio-containing compounds F12 and F15 may be of interest for further development of furanone- based inhibitors of bacterial biofilms. However, the potential mutagenicity of these furanones revealed in the Ames test serves as a contraindication for their direct application and requires further modification of their structure.

## Abbreviations

MIC: minimum inhibitory concentration
MBIC: minimum biofilm inhibitory concentration
